# Supplementary material for: Boosting quantum yields in two-dimensional semiconductors via proximal metal plates
Source: Nat Commun. 2021 Dec 7;12:7095. doi: 10.1038/s41467-021-27418-x (PMC8651657; doi:10.1038/s41467-021-27418-x)
Supplement: Supplementary file 1 — Supplementary Information [file 41467_2021_27418_MOESM1_ESM.pdf]

Supplementary Information

Boosting quantum yields in 2D semiconductors via proximal metal plates

Yongjun Lee[1,§], Johnathas D'arf Severo Forte[2, §], Andrey Chaves[2,3, §], Anshuman Kumar[4], Trang Thu Tran[1], Youngbum Kim[1], Shrawan Roy[1], Takashi Taniguchi[5], Kenji Watanabe[6], Alexey Chernikov[7], Joon I. Jang[8], Tony Low[9,] and Jeongyong Kim[1,*]*

[1]Department of Energy Science, Sungkyunkwan University, Suwon 16419, Republic of Korea

[2]Departamento de Física, Universidade Federal do Ceará, Campus do Pici, 60455-900 Fortaleza, Ceará, Brazil

[3]Department of Physics, University of Antwerp, Groenenborgerlaan 171, B-2020 Antwerpen, Belgium

[4]Physics Department, Indian Institute of Technology Bombay, Mumbai 400076, India

[5]International Center for Materials Nanoarchitectonics, National Institute for Materials Science, 1-1 Namiki, Tsukuba 305-0044, Japan

[6]Research Center for Functional Materials, National Institute for Materials Science, 1-1 Namiki, Tsukuba 305-0044, Japan

[7]Department of Physics, University of Regensburg, Regensburg, D-93040, Germany

[8]Department of Physics, Sogang University, Seoul 04107, Republic of Korea

[9]Department of Electrical & Computer Engineering, University of Minnesota, Minneapolis, MN 55455, USA

AUTHOR INFORMATION

[§]These authors contributed equally: Yongjun Lee, Johnathas D'arf Severo Forte, Andrey Chaves

Corresponding Authors

* Tony Low (tlow@umn.edu) and Jeongyong Kim (j.kim@skku.edu)

(1) Optical interference effect on the PL intensity

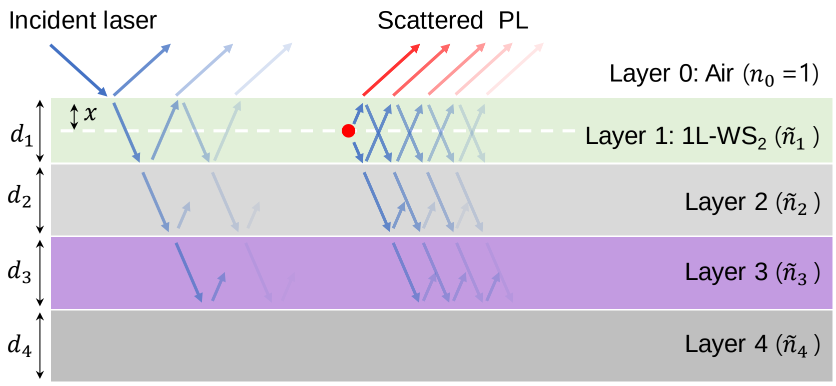

Supplementary Figure 1. Schematic diagram of the optical interference effect in a five-layered system. (Left: absorption and right: PL emission processes) The schematic is modified from Ref. 1.

The optical interference effect can affect the observed photoluminescence (PL) intensity measured by the microscope due to multiple reflections of the light from layer interfaces [1-4]. Many researchers have considered the optical interference effect of multiple layered substrates to explain different contrast or PL intensities of two-dimensional (2D) thin films such as graphene and 2D transition metal dichalcogenides (TMDs) [1-6]. Due to the multiple reflections of the light between the different dielectrics, there are multiple chances to absorb the excitation light and scatter the PL from monolayer (1L)-WS$_2$. The net absorption of excitation light and scattering of PL from 1L-WS$_2$ in the four-layered system can be expressed as [1]

$$F_{abs} = t_1 \frac{\left[1 + r_2 r_3 e^{-2i\beta_2}\right] e^{-i\beta_x} + \left[r_2 + r_3 e^{-2i\beta_2}\right] e^{-i(2\beta_1 - \beta_x)}}{1 + r_2 r_3 e^{-2i\beta_2} + (r_2 + r_3 e^{-2i\beta_2}) r_1 e^{-2i\beta_1}}, \quad (1)$$

$$F_{sc} = t_1' \frac{\left[1 + r_2 r_3 e^{-2i\beta_2}\right] e^{-i\beta_x} + \left[r_2 + r_3 e^{-2i\beta_2}\right] e^{-i(2\beta_1 - \beta_x)}}{1 + r_2 r_3 e^{-2i\beta_2} + (r_2 + r_3 e^{-2i\beta_2}) r_1 e^{-2i\beta_1}}, \quad (2)$$

where $t_1 = 2n_0/(\tilde{n}_1 + n_0)$, $t'_1 = 2\tilde{n}_1/(\tilde{n}_1 + n_0)$, $r_n = (\tilde{n}_{n-1} - \tilde{n}_n)/(\tilde{n}_{n-1} + \tilde{n}_n)$ are the Fresnel transmission and reflection coefficients at normal incidence for the interfaces between different layers having their refractive indices that include air (n_0=1) and the 2D layer i (n_i) (by Equation editor). Here, the prime in the transmission coefficient corresponds to the internal case, i.e., transmission from the 2D layer to air. The phase differences in Eqs. (1) and (2) are defined as $\beta_x = 2\pi x\tilde{n}_1/\lambda$ and $\beta_i = 2\pi d_i\tilde{n}_i/\lambda$, where x is a point in the depth of 1L-WS_2 (layer 1) in which both absorption and emission occur, d_i is the thickness of the layer i (the thickness of 1L-WS_2; d_1 is taken to be 0.7 nm) and λ is the wavelength of light (532 nm for excitation and 615 nm for the PL of 1L-WS_2). For three- or five-layered system (the case for a suspended sample or air/1L-WS_2/hBN/SiO_2/Si), r_3 in Eqs. (1) and (2) is 0 or should be replaced by $(r_3 + r_4e^{-2i\beta_3})/(1 + r_3r_4e^{-2i\beta_3})$, respectively.

The net emission intensity of 1L-WS_2 on the substrate (I_{sub}) can be given by

$$I_{sub} = \int_0^{d_1} |F_{abs} \cdot F_{sc}|^2 dx. \quad (3)$$

We also calculated the net emission intensity of the suspended 1L-WS_2 in air (I_{sus}). We defined the enhancement factor (EF) as the ratio of calculated values for various substrates (I_{sub}) and that for the suspended 1L-WS_2 (I_{sus}), I_{sub}/I_{sus}[2].

The calculated EF for the quartz substrate, 300-nm-thick SiO_2 on the Si substrate (SiO_2/Si) and 27-nm-thick hBN on SiO_2/Si (used in Fig. 2a in the main text) are 0.396, 1.901 and 0.527, respectively. The EF as a function of hBN thickness on Au is shown in Fig. 1e (red line) in the main text. A higher EF value on a typical substrate means that 1L-WS_2 would have a higher PL intensity on it under the same excitation conditions even if the samples have the same quantum yields (QYs). The complex refractive indices of the materials that were used for the calculations

are given in Supplementary Table 1.

Supplementary Table 1. Details of refractive indices of the materials used for the calculations.

Materials	Wavelength (nm)	Indices (n-ik)		References
		n	k	
WS_2	532 (excitation)<br>615 (emission)	4.9<br>4.9	0.9<br>1.8	7
Quartz	532<br>615	1.54	0	8
SiO_2	532<br>615	1.46<br>1.45	0<br>0	9
Si	532<br>615	4.15<br>3.9	0.05<br>0.02	10
Au	532<br>615	0.55<br>0.23	2.2<br>3.1	11
hBN	532<br>615	1.85	0<br>0	12

(2) Variation in absorption as a function of hBN thickness on the Au plate

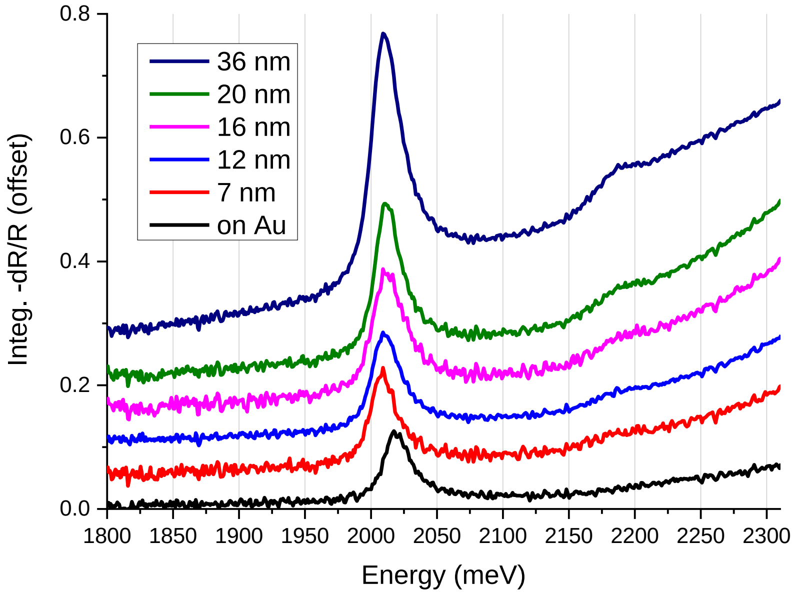

Supplementary Figure 2. Differential reflectance spectra obtained from 1L-WS_2 on different thicknesses of hBN on Au. Increasing absorption with increasing hBN thickness is clearly shown.

(3) PL spectra and images of hBN-encapsulated 1L-WS_2 at low and high exciton densities

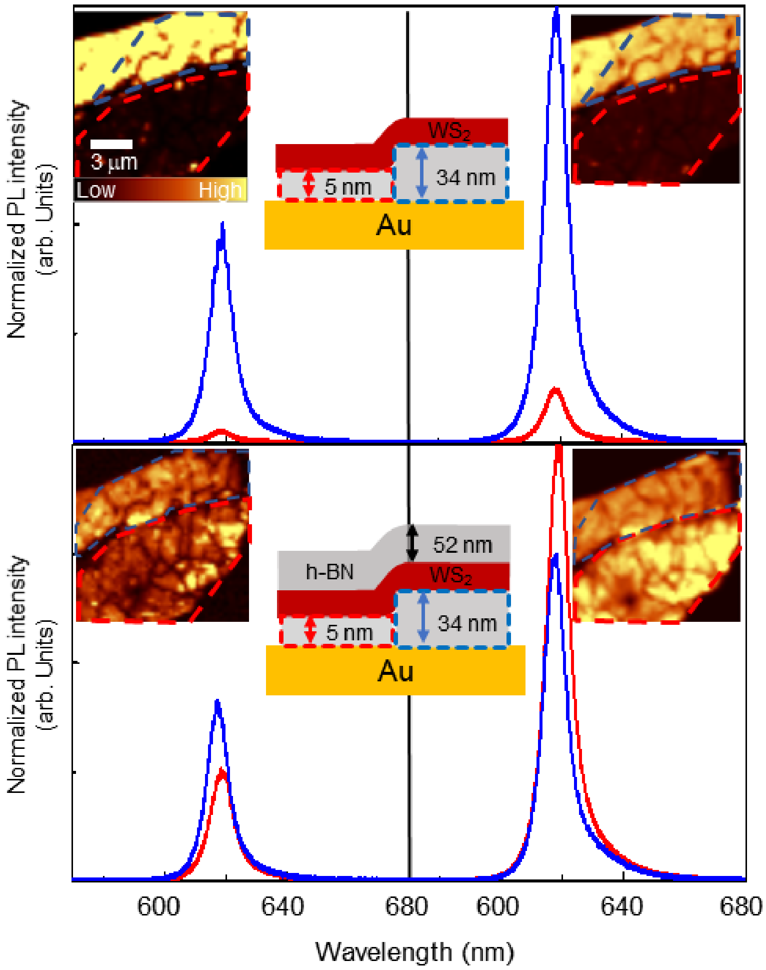

Supplementary Figure 3. Observed PL spectra and intensity images of 1L-WS_2 on hBN/Au with (upper panels) and without (lower panels) top 52 nm hBN, obtained at low (left panels) and high (right panels) excitation powers. Insets: PL intensity images of 1L-WS_2 on 5 nm hBN and 34 nm hBN on the Au substrate. Contrast is normalized for each image.

(4) Experimental estimation of the EEA rate

The solution to Eq. (1) in the main text is given by [13,14]

$$n_{ex}(t) = \frac{n_{ex}^0}{\exp\left(\frac{t}{\tau_0}\right)(1 + n_{ex}^0 \gamma \tau_0) - n_{ex}^0 \gamma \tau_0}, (4)$$

where n_{ex}^0 is the initial exciton density and all the other parameters are defined in the main text. With some assumptions, Eq. (4) could be simplified as explained below.

1) In the case of $n_{ex}^0 \ll (\gamma\tau_0)^{-1}$, the exciton density is not high enough to cause EEA. Therefore, the exciton transient shows linear decay with an exciton lifetime τ_0 and Eq. 4 can be simplified to $n_{ex}(t) = n_{ex}^0 \exp(-t/\tau_0)$.

Supplementary Table 2. Summary of the parameters for EEA extraction.

Substrates	hBN thickness (nm)	Lifetime, τ_0 (ns)	EEA rate, γ (cm^2s^{-1})	n_{ex}^0 ($\times 10^{10}$ cm^{-2}) Min.	n_{ex}^0 ($\times 10^{10}$ cm^{-2}) Max.	$(\gamma\tau_0)^{-1}$ ($\times 10^{10}cm^{-2}$)
hBN/Au	0	0.81	0.05426	3.1	11.1	2.05
	7	0.93	0.01776	44	202	6.03
	16	0.98	0.03599	72	329	2.83
	20	0.86	0.02185	82	374	5.55
	36	1.23	0.04885	67	307	1.66
SiO_2		0.94	0.12643	15	83	0.84

2) In the case of $n_{ex}^0 \gg (\gamma\tau_0)^{-1}$, we need to consider the temporal resolution of the detector. For example, when the temporal bin is very short $t \ll (\gamma n_{ex}^0)^{-1}$ Eq. (4) can be simplified to $n_{ex}(t) = n_{ex}^0/(1 + n_{ex}^0 \gamma t)$, which is commonly used for the estimation of the EEA rate using a fast detection system having a temporal resolution

below 100 ps [15,16]. However, if it is not sufficiently short $t \gg (\gamma n_{ex}^0)^{-1}$ because of experimental limitation, the exciton decay is given by $n_{ex}(t) = (\gamma\tau_0)^{-1}\exp(-t/\tau_0)$ [13].

We summarized all the parameters used in Supplementary Table 2. The series of TRPL transients and $n_{ex}^{-1}(t)\tau_0^{-1} = \gamma\exp(t/\tau_0)$ were displayed in Supplementary Fig. 4. For all the cases, the initial exciton density satisfies well the condition for the assumption of $n_{ex}^0 \gg (\gamma\tau_0)^{-1}$. In case of direct Au contact, the initial exciton density is higher than $(\gamma\tau_0)^{-1}$ only by a factor of 1.4~5. Such a low initial exciton density of the TRPL measurement from the specimen on Au suggests that the EEA rate quoted in the main text could have been somewhat overestimated.

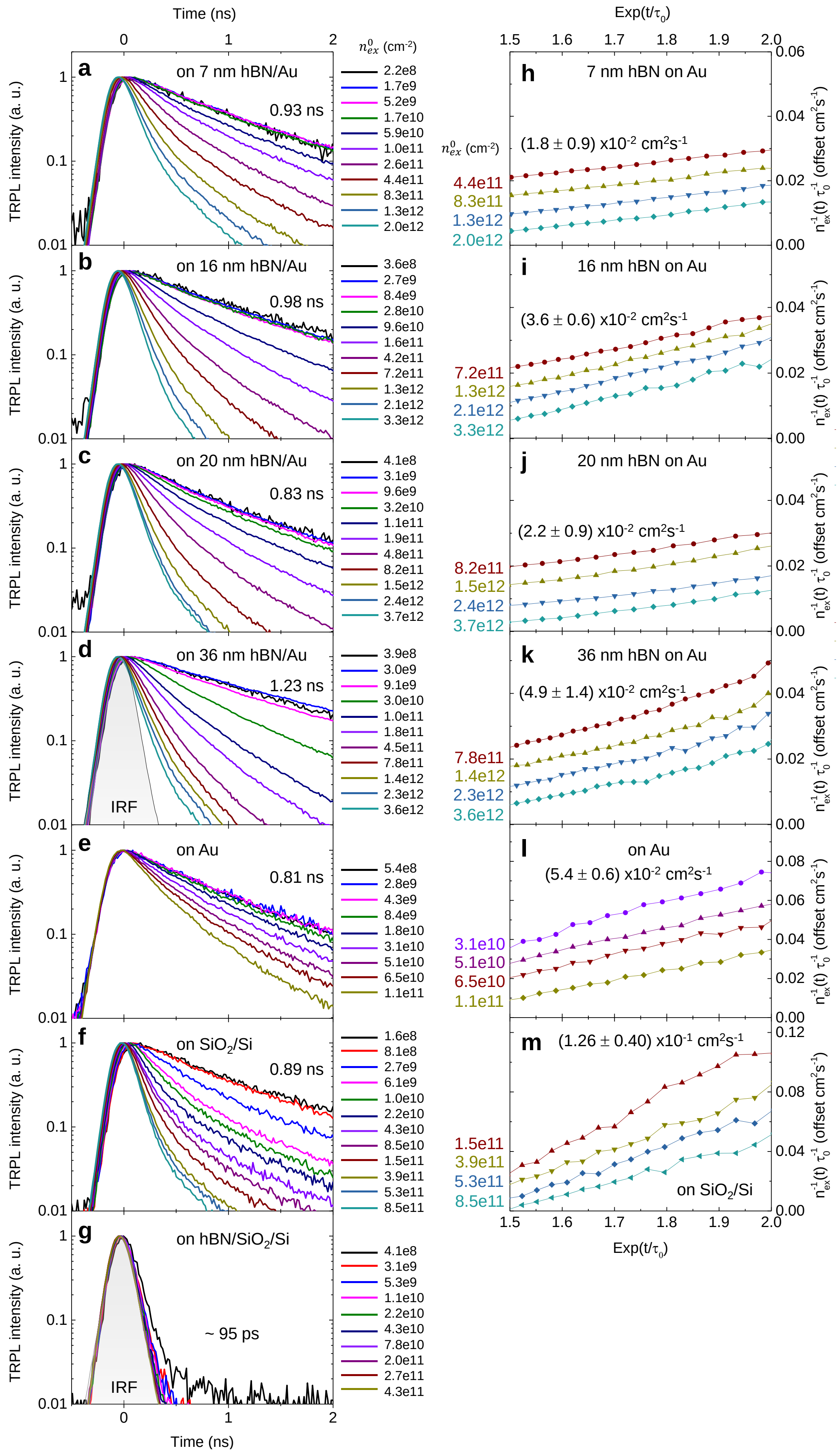

Time (ns)
TRPL intensity (a. u.)
a on 7 nm hBN/Au 0.93 ns
b on 16 nm hBN/Au 0.98 ns
c on 20 nm hBN/Au 0.83 ns
d on 36 nm hBN/Au 1.23 ns
IRF
e on Au 0.81 ns
f on SiO2/Si 0.89 ns
g on hBN/SiO2/Si ~ 95 ps
Exp(t/τ0)
h 7 nm hBN on Au (1.8 ± 0.9) x10-2 cm2s-1
i 16 nm hBN on Au (3.6 ± 0.6) x10-2 cm2s-1
j 20 nm hBN on Au (2.2 ± 0.9) x10-2 cm2s-1
k 36 nm hBN on Au (4.9 ± 1.4) x10-2 cm2s-1
l on Au (5.4 ± 0.6) x10-2 cm2s-1
m (1.26 ± 0.40) x10-1 cm2s-1 on SiO2/Si
n-1ex(t) τ0-1 (offset cm2s-1)

Supplementary Figure 4. TRPL transient series and EEA estimation. a-g. TRPL decays and **h-m**. Plot of $n_{ex}^{-1}(t)\tau_0^{-1}$ vs. exp (t/τ_0) series at each hBN thickness condition. The values of lifetimes (EEA rates) are given in **a-g** (**h-m**). The EEA rate of 1L-WS_2 on the hBN/SiO_2/Si substrate could not be extracted since the corresponding lifetime is too short to be distinguished from instrument response function (IRF). Note: The degree of absorption varies with different substrate configuration, resulting in different initial exciton densities.

(5) Estimation of the absolute quantum yield (a-QY) using a microscope

The integrating sphere was used for the estimation of the absolute QY (a-QY) of the samples of macroscopic sizes. In case of 1L-TMDs, however, measurements using a microscope are required due to the microscopic sizes of the samples. The details of a-QY measurement are explained in Ref. [17].

1) a-QY measurement by an integrating sphere

We fabricated a poly methyl methacrylate (PMMA) thin film embedded with rhodamine 6G (R6G) as reference samples using spin coating on quartz substrates[17]. The PMMA/R6G samples are suitable for the a-QY measurement using an integrating sphere (819C-SL-3.3, Newport) because it has homogeneous light emission on a centimeter scale with thickness of 80 or 300 nm (using PMMA C2 or C4, respectively). Each of the PMMA/R6G films was loaded into the integrating sphere and excited using a 532 nm laser. The scattered laser light and the PL from the film were guided to a monochromator equipped with a charge-coupled device camera through an optical fiber connected to the integrating sphere. By comparing the intensities of the absorbed 532 nm laser line and the emitted PL from the PMMA/R6G films, the a-QY values of the PMMA/R6G films were estimated. These a-QY values were double-checked using a commercial a-QY spectrometer (Quantaurus-QY, Hamamatsu Photonics) as well.

2) a-QY measurement under a microscope

To estimate the a-QY of 1L-WS_2, we exfoliated and transferred 1L-WS_2 flakes on quartz substrates with the same configuration as PMMA/R6G films. We then measured and calculated PL and absorption of the PMMA/R6G films and 1L-WS_2 under the same confocal microscope and detection configurations.

The values of absorption (A) can be estimated using reflectance or transmittance measurements with thin film approach boundary conditions using the following relation [18,19]:

$$\frac{\Delta R}{R} \cong \frac{4}{n_{sub}^2 - 1} A_{free\ standing}$$

$$\frac{\Delta T}{T} \cong -\frac{2}{n_{sub} + 1} A_{free\ standing}$$

(5)

Because our PMMA/R6G film and 1L-WS_2 are placed on quartz substrates for a-QY measurements, Eg. (5) is modified to

$$\frac{\Delta R}{R} \cong \frac{n_{sub} + 1}{n_{sub} - 1} A$$

$$\frac{\Delta T}{T} \cong -\frac{n_{sub} + 1}{2} A$$

(6)

By comparing absorption and PL counts of 1L-WS_2 and the PMMA/R6G films, we were able to estimate the a-QY of 1L-WS_2.

(6) Theoretical model for exciton-exciton annihilation and interactions.

1) Exciton-exciton Auger scattering

Let us consider the Auger process where the interaction between conduction band electrons from two excitons with momenta $\mathbf{K}_1$ and $\mathbf{K}_2$ induce a nonradiative recombination event where the energy of the recombined exciton is used to ionize the electron-hole pair of the other exciton. In this case, we start with an initial state $|i\rangle = |X_1\rangle|X_2\rangle$composed of two excitons and end up with a final state $|f\rangle = |\mathbf{k}_e\, \mathbf{k}_h\rangle$ composed of a single electron and a hole with momenta $\mathbf{k}_e$and $\mathbf{k}_h$, respectively[20-22].

The scattering rate for this process is obtained according to Eq. (4) of the main manuscript, which is obtained assuming electrons and holes with the same effective mass $m_e = m_h = 0.3m_0$, which is a fairly good approximation for 1L-WS_2 [23,24] that simplifies the analytical calculations.

The scattering matrix for this direct Auger process is given by

$$\mathbf{M}_{dir} = \langle i|V|f\rangle = \sum_{\mathbf{K}_1,\mathbf{K}_2} \delta_{\mathbf{K}_f,\mathbf{K}_1+\mathbf{K}_2} \frac{\Phi_{1s}(0)\gamma_3}{E_g} \left[V(K_1)I(K_1)\left(K_{1x} + iK_{1y}\right) + V(K_2)I(K_2)\left(K_{2x} + iK_{2y}\right)\right], \quad (7)$$

where $V(K)$ is the screened electron-electron interaction potential given by Eq. (2) of the main text in reciprocal space, $\Phi_{1s}(r)$ is the 1s exciton wave function, E_g is the quasi-particle gap, γ_3 is a band structure parameter [25],

and the Kronecker delta function guarantees momentum conservation. The integral terms $I(K)$ involving bound and unbound ($\Phi_{k_f,l}$) exciton wave functions are approximately

$$I(K) = \int e^{i\mathbf{K}\cdot\mathbf{r}/2}\, \Phi^*_{k_f,l}\Phi_{1s} d\mathbf{r} \propto \frac{K}{k_f^3 a_B^2}, \quad (8)$$

where a_B is the exciton Bohr radius [20]. Notice that in Eqs. (7) and (8) we have re-written $\mathbf{k}_e$and $\mathbf{k}_h$in terms of the relative momentum $\mathbf{k}_f$ and center-of-mass momentum $\mathbf{K}_f$ of the final

electron-hole pair.

The kinetic energy of the exciton is typically much smaller than the energy gap and binding energies, which allows us to approximate the relative momentum, from the energy conservation term in Eq. (4) of the main manuscript, by

$$k_f = \sqrt{\frac{(E_g - 2E_B)M}{2\hbar^2}} \tag{9}$$

Substituting Eqs. (8) and (9) in Eq. (7) and performing the summation, we obtain the matrix element $\mathbf{M}_{dir}$. Our calculation for the EEA rate constant using $\mathbf{M}_{dir}$ and Eq. (4) is in shown as a function of hBN thickness in Fig. 5 of the main text.

2) Exciton-exciton interaction potential

The proximity with the metal is also expected to strongly affect exciton-exciton interactions. In order to calculate the interaction between a pair of excitons, we consider the Hamiltonian

$$H_{XX} = -\frac{\hbar^2\nabla_1^2}{2\mu} - \frac{\hbar^2\nabla_2^2}{2\mu} + V(r_1) + V(r_2) + V_{XX}(\mathbf{r}_1, \mathbf{r}_2, R), \tag{10}$$

where the original eight coordinates of the two electrons and two holes were converted into relative electron-hole coordinates of the first and second excitons, $\mathbf{r}_1$ and $\mathbf{r}_2$ respectively, a relative coordinate representing the distance between the two excitons R, and a center-of-mass coordinate for the whole system. The kinetic energy of the latter is a good quantum number and can be neglected. Therefore, we are eventually left with the four-dimensional Hamiltonian H_{XX}, with a parameter R, in Eq. (10).

For a given distance R between the two excitons, we estimate the energy of the pair of excitons by using a wave function of the form

$$\Phi_{XX}(r_1, r_2, R) = \Phi_{1s}(r_1)\Phi_{1s}(r_2)[1 - V_{XX}(r_1, r_2, R)/E_B] \tag{11}$$

and performing the integral of Eq. (11) numerically. This trial wave function is inspired in the variational function used in the derivation of the van der Waals interaction potential between two hydrogen atoms, see e.g. Ref. [26]. In fact, it converges exactly to the hydrogenic case as we replace V_{XX} by the corresponding Coulomb potential, thus leading to a van der Waals interaction between excitons in that limit.

We are interested in the average exciton-exciton interaction energy. In order to estimate this quantity, we assume pairwise interactions between excitons that are separated by a distance roughly given by $R = 1/\sqrt{n}$, where n is the exciton density.

Interestingly, our numerical results for the exciton pair interaction energy as a function of hBN thickness as a spacer between 1L-WS_2 and the Au substrate reveal a similar trend as compared to the experimentally observed EEA rate constant, as shown in Fig. 5b of the main text, which suggests that the screening due to the metal affects both quantities in a similar manner.

Reference

1 Yoon, D. *et al.* Interference effect on Raman spectrum of graphene on SiO2 /Si. *Physical Review B - Condensed Matter and Materials Physics* **80**, 1–6 (2009).

2 Buscema, M., Steele, G. A., van der Zant, H. S. J. & Castellanos-Gomez, A. The effect of the substrate on the Raman and photoluminescence emission of single-layer MoS2. *Nano Research* **7**, 561–571 (2014).

3 Wang, Y. Y., Ni, Z. H., Shen, Z. X., Wang, H. M. & Wu, Y. H. Interference enhancement of Raman signal of graphene. *Applied Physics Letters* **92**, (2008).

4 Ding, L. *et al.* Understanding Interlayer Coupling in TMD-hBN Heterostructure by Raman Spectroscopy. *IEEE Transactions on Electron Devices* **65**, 4059–4067 (2018).

5 Roddaro, S., Pingue, P., Piazza, V., Pellegrini, V. & Beltram, F. The optical visibility of graphene: Interference colors of ultrathin graphite on SiO2. *Nano Letters* **7**, 2707–2710 (2007).

6 Blake, P. *et al.* Making graphene visible. *Applied Physics Letters* **91**, (2007).

7 Jung, G. H., Yoo, S. J. & Park, Q. H. Measuring the optical permittivity of twodimensional materials without a priori knowledge of electronic transitions. *Nanophotonics* **8**, 263–270 (2018).

8 Ghosh, G. Dispersion-equation coefficients for the refractive index and birefringence of calcite and quartz crystals. *Optics Communications* **163**, 95–102 (1999).

9 Tan, C. Z. Determination of refractive index of silica glass for infrared wavelengths by IR spectroscopy. *Journal of Non-Crystalline Solids* **223**, 158–163 (1998).

10 Aspnes, D. E. & Studna, A. A. Dielectric functions and optical parameters of Si, Ge, GaP, GaAs, GaSb, InP, InAs, and InSb from 1.5 to 6.0 eV. *Physical Review B* **27**, 985–1009 (1983).

11 P. B. Johnson and R. W. Christy. Optical Constant of the Nobel Metals. *Physical Review B* **6**, 4370–4379 (1972).

12 Golla, D. *et al.* Optical thickness determination of hexagonal boron nitride flakes. *Applied Physics Letters* **102**, (2013).

13 Yuan, L. & Huang, L. Exciton dynamics and annihilation in WS 2 2D semiconductors. *Nanoscale* **7**, 7402–7408 (2015).

14 Kulig, M. *et al.* Exciton Diffusion and Halo Effects in Monolayer Semiconductors. *Physical Review Letters* **120**, 207401 (2018).

15 Sun, D. *et al.* Observation of Rapid Exciton–Exciton Annihilation in Monolayer Molybdenum Disulfide. *Nano Letters* **14**, 5625–5629 (2014).

16 Yu, Y. *et al.* Fundamental limits of exciton-exciton annihilation for light emission in transition metal dichalcogenide monolayers. *Physical Review B* **93**, 2–6 (2016).

17 Roy, S. *et al.* Measurement of quantum yields of monolayer tmds using dye-dispersed pmma thin films. *Nanomaterials* **10**, (2020).

18 Aslan, O. B. Probing Transition Metal Dichalcogenides via Strain-Tuned and Polarization-Resolved Optical Spectroscopy. (2017).

19 Li, Y. & Heinz, T. F. Two-dimensional models for the optical response of thin films. *2D Materials* **5**, (2018).

20 Han, B. *et al.* Exciton States in Monolayer MoSe2 and MoTe2 Probed by Upconversion Spectroscopy. *Physical Review X* **8**, 1–17 (2018).

21 Kavoulakis, G. M. & Baym, G. Auger decay of degenerate and Bose-condensed excitons in Cu2O. *Physical Review B* **54**, 16625–16636 (1996).

22 Wang, F., Wu, Y., Hybertsen, M. S. & Heinz, T. F. Auger recombination of excitons in one-dimensional systems. *Physical Review B - Condensed Matter and Materials Physics* **73**, 1–5 (2006).

23 Haastrup, S. *et al.* The Computational 2D Materials Database: High-throughput modeling and discovery of atomically thin crystals. *2D Materials* **5**, (2018).

24 Gjerding, M. N. *et al.* Recent progress of the computational 2D materials database (C2DB). *2D Materials* **8**, (2021).

25 Kormányos, A. *et al.* Corrigendum: k.p theory for two-dimensional transition metal dichalcogenide semiconductors (2015 2D Mater. 2 022001). *2D Materials* **2**, 049501 (2015).

26 Schiff, L. I. *Quantum mechanics*. (McGraw-Hill, 1949).
